# [Retracted] miR‑148a‑3p suppresses epithelial ovarian cancer progression primarily by targeting c‑Met

**DOI:** 10.3892/ol.2024.14644

**Published:** 2024-08-27

**Authors:** Wen Wang, Jing Dong, Maoxiu Wang, Shujuan Yao, Xiangyu Tian, Xiujuan Cui, Shijie Fu, Shiqian Zhang

Oncol Lett 15: 6131–6136, 2018; DOI: 10.3892/ol.2018.8110

Following the publication of this paper, it was drawn to the Editor's attention by a concerned reader that the invasion assay data shown in Fig. 2C on p. 6134 had already appeared in an article written by different authors that had already been submitted for publication [Li P, Wang H, Hou M, Li D and Bai H: Upstream stimulating factor 1 (USF1) enhances the proliferation of glioblastoma stem cells mainly by activating the transcription of mucin 13 (MUC13). Pharmazie 72: 98-102, 2017].

Owing to the fact that the contentious data in the above article had already been submitted for publication prior to its submission to *Oncology Letters*, the Editor has decided that this paper should be retracted from the Journal. The authors were asked for an explanation to account for these concerns, but the Editorial Office did not receive a reply. The Editor apologizes to the readership for any inconvenience caused.

